# A short-term statin treatment changes the contractile properties of fast-twitch skeletal muscles

**DOI:** 10.1186/s12891-016-1306-2

**Published:** 2016-10-28

**Authors:** Antoine Boulanger Piette, Sébastien S. Dufresne, Jérôme Frenette

**Affiliations:** 1 Centre Hospitalier Universitaire de Québec, Centre de Recherche du Centre Hospitalier de l’Université Laval (CHUQ-CHUL), Axe Neurosciences, Université Laval, Quebec City, QC G1V 4G2 Canada; 2 Département de Réadaptation, Faculté de Médecine, Université Laval, Quebec City, QC G1V 0A6 Canada

**Keywords:** Statin, Myopathy, Muscle, Contractile, Cholesterol

## Abstract

**Background:**

Cumulative evidence indicates that statins induce myotoxicity. However, the lack of understanding of how statins affect skeletal muscles at the structural, functional, and physiological levels hampers proper healthcare management. The purpose of the present study was to investigate the early after-effects of lovastatin on the slow-twitch *soleus* (Sol) and fast-twitch *extensor digitorum longus* (EDL) muscles.

**Methods:**

Adult C57BL/6 mice were orally administrated with placebo or lovastatin [50 mg/kg/d] for 28 days. At the end of the treatment, the isometric ex vivo contractile properties of the Sol and EDL muscles were measured. Subtetanic and tetanic contractions were assessed and contraction kinetics were recorded. The muscles were then frozen for immunohistochemical analyses. Data were analyzed by two-way ANOVA followed by an *a posteriori* Tukey’s test.

**Results:**

The short-term lovastatin treatment did not induce muscle mass loss, muscle fiber atrophy, or creatine kinase (CK) release. It had no functional impact on slow-twitch Sol muscles. However, subtetanic stimulations at 10 Hz provoked greater force production in fast-twitch EDL muscles. The treatment also decreased the maximal rate of force development (dP/dT) of twitch contractions and prolonged the half relaxation time (1/2RT) of tetanic contractions of EDL muscles.

**Conclusions:**

An early short-term statin treatment induced subtle but significant changes in some parameters of the contractile profile of EDL muscles, providing new insights into the selective initiation of statin-induced myopathy in fast-twitch muscles.

## Background

Statins are a class of drugs used to treat hypercholesterolemia by inhibiting HMG-CoA reductase, which is a rate-limiting step in the biosynthesis of cholesterol. Statins are effective for the primary and secondary prevention of cardiovascular diseases, but their use is frequently associated with statin-induced myopathy (SIM). SIM encompasses myalgia, cramps, weakness, and even rhabdomyolysis [1–3]. While the pathophysiology of SIM is heterogeneous and poorly understood, it appears to be multifactorial and may involve multiple pathophysiological defects, including genetic predisposition [4], early mitochondrial dysfunctions [5–8], prenylated proteins [9], the inhibition of protein geranylgeranylation [10], ubiquinone depletion [11, 12], monocarboxylate transporter 4 expression [13, 14], loss of t-tubular region architecture [15, 16], altered membrane fluidity [17], and immune-mediated necrotis [18].

Electrical stimulation causes Ca^2+^ release from the sarcoplasmic reticulum (SR), triggering actin-myosin cross-bridging and muscle contraction. Ca^2+^ is then rapidly transported back into the SR lumen by SERCA [sarco (endo) plasmic reticulum Ca^2+^ATPase], an ATP-dependent Ca^2+^ pump, inducing muscle relaxation and preparing the muscle for the next contraction. Myoplasmic Ca^2+^ also regulates protein synthesis, protein degradation, and fiber phenotype by controlling Ca^2+^-sensitive proteases, transcription factors, and mitochondrial adaptations. Mammalian skeletal muscles contain two SERCA isoforms. SERCA-1a is expressed in fast-twitch myofibers while SERCA-2a is usually limited to slow-twitch myofibers [19]. SERCA pumps are defective in several forms of muscle and heart disease, prolonging muscle relaxation and reducing muscle force production [19, 20]. Impairment of the sarcoplasmic reticulum (SR)/mitochondria Ca^2+^ release system and the concomitant loss of Ca^2+^ homeostasis may also be a crucial event leading to SIM [21–23]. A rise in myoplasmic Ca^2+^ at rest triggers the calmodulin/calcineurin/Ca^2+/^calmodulin-dependent protein kinase II pathway, activating NFATc1 and PGC-1α, two transcription factors and modulators that facilitate the oxidative machinery and slow-twitch phenotype [24, 25]. Interestingly, fast-twitch myofibers, which contain three times more Ca^2+^ in the SR than slow-twitch myofibers, are the first to disappear in numerous forms of muscle disease and aging [26–28]. These fibers appear to be especially prone to SIM-induced dysfunction and necrosis [29–31]. The failure to preserve physiological resting cytosolic Ca^2+^ concentrations in SIM may potentially activate the calpain Ca^2+−^dependant proteolytic pathway [21], repress protein synthesis [32, 33], and stimulate the ubiquitin-proteasome system [34, 35], favoring atrophy [33], myocellular damage [34] and, ultimately, apoptosis [36, 37].

Since statins are widely prescribed and since SIM may lead to the discontinuation of treatment, sometimes after less than 2 weeks, we investigated the functional impact of a short-term lovastatin treatment (28 days) on the ex vivo contractile properties of slow-twitch Sol and fast-twitch EDL muscles We also investigated the impact of a short-term lovastatin treatment on muscle integrity, serum creatine kinase release, and myosin phenotype in fast-twitch EDL muscles. We showed that lovastatin caused subtle early-stage functional changes in EDL muscles but not in Sol muscles. The functional changes were not accompanied by morphological or phenotypic changes.

## Methods

### Animals and experimental design

Male C57BL/6 mice (30–35 g) obtained from Charles River Laboratories International (St-Constant, QC, Canada) were housed one per cage, maintained in a 12 h light/dark cycle, and given food and water ad libitum. The mice were orally administered with placebo gelatin gel or lovastatin [50 mg/kg/day; J&K scientific, Shanghai, China] for 28 days (*n* = 8). Lovastatin was chosen for its lipophilic nature, mid-range potency and clinical relevance. The dosage is commonly used and was selected because the HMG-CoA reductase activity and pharmacokinetics of lovastatin are different in rodents and humans [38–40]. The Animal Care and Use Committee of the CHUQ Research Centre approved all the experiments, which complied with Canadian Council on Animal Care guidelines.

### Isometric contractile properties

After a 28-day lovasatin treatment and 24 h after the last dose of lovastatin, the mice were injected with buprenorphine [0.1 mg/kg] for analgesia and pentobarbital sodium [50 mg/kg] for anesthesia. The depth of analgesia/anesthesia was monitored by pinching the Achilles tendon. The Sol and EDL muscles were completely resected from the hindlimb, attached to a lever arm system (305B-LR dual-mode; Aurora Scientific, Canada) controlled by dynamic muscle control and data acquisition software (Aurora Scientific Inc., Aurora, ON, Canada), and incubated at 25 °C in an oxygenated Krebs-Ringer solution supplemented with 2 mg/mL of glucose [41, 42]. During the ex-vivo equilibrium period, contralateral muscles were collected and the mice were euthanized by cervical dislocation under anesthesia. Once the optimal length (Lo) and twitch contractions had been determined, the muscles were stimulated for 500 ms at 10, 20, 50, 80, 100, or 120 Hz to induce subtetanic and tetanic contractions. Time-to-peak twitch tension (TPT, ms), half relaxation time (1/2 RT), (ms), twitch tension (Pt, g), maximum tetanic tension (P_0_, g), and peak rate of tension development (dP/dT) values were also recorded. At the end of the contractile property measurements, the lengths of the muscles were measured. The tendons were removed, and the muscles were weighed, embedded and frozen in isopentane cooled in liquid nitrogen, and stored at −80 °C until used for the immunohistochemical assays. The mice were then euthanized by cervical dislocation under anesthesia.

### Enzymatic assays and immunohistochemical experiments

Serum creatine kinase activity was determined using a colorimetric assay kit (BioVision, USA). To assess muscle atrophy, the Sol and EDL muscles were perpendicularly sectioned (10 μm), stained with hemotoxylin and eosin (H&E; Sigma-Aldrich, USA), and observed using an inverted light microscope, (Nikon, Canada). Cross-sectional areas (CSA) were analyzed using ImageJ software 1.46r (NIH, USA). Myosin heavy chain (MyHC) I and MyHC II antibodies (Leica Biosystems, Canada) were used to determine changes in fast and slow phenotypes in EDL muscles. Approximately 100 myofibers per muscle were quantified. All values are expressed as means ± standard errors of the mean. Data were analyzed by two-way ANOVA (InStat software, v.3). When a significant F ratio was obtained, an *a posteriori* Tukey’s test was performed. The level of significance was set at *p* < 0.05.

## Results

**Fig. 1:**
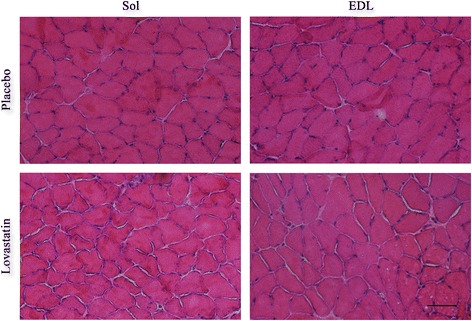
Cross-sections of *soleus* and *extensor digitorum longus* muscles stained with hematoxylin and eosin. There were no significant differences in the structure and histologic features of Sol and EDL muscles following placebo or lovastatin [50 mg/kg/day] treatments. Scale bar = 100 μm

To investigate the effect of lovastatin on muscle atrophy, the masses and CSA of Sol and EDL muscles from placebo and statin-treated mice were determined. The 28-day statin treatment had no significant effect on muscle mass or CSA values (Tables 1 and 2). Serum creatine kinase levels, which are a biomarker of muscle damage, were below the limit of detection. Hematoxylin and eosin (H&E) staining revealed no significant muscle damage in the control and statin-treated mice (Fig. 1).

**Table 1:** Morphology and contractile properties of Sol muscles

Measure	Placebo	Lovastatin
Muscle mass; (mg)	10,79 ± 0,10	10,92 ± 0,76
Cross-sectional area; (μm^2^)	1101,98 ± 78,55	1146,79 ± 88,46
Twitch tension Pt; (g)	3,25 ± 0,28	3,81 ± 0,51
Maximal tetanic force P_0_; (g)	22,72 ± 1,11	25,72 ± 0,45 ^*p*=0.053^
Specific force sP_0_; (g/mg)	2,18 ± 0,09	2,38 ± 0,09
Pt/P_0_	0,14 ± 0,01	0,14 ± 0,02
Time to peak tension TPT; (ms)	52,14 ± 2,15	47,83 ± 3,28
1/2 RT at Pt; (ms)	47,14 ± 2,59	47,01 ± 2,30

The treatment with lovastatin [50 mg/kg/day] for 28 days had the tendency (0,05 < *p* < 0,06) to increase the maximum tetanic force (P_0_) of Sol muscles. Data are expressed as means ± SE, *n* = 8 mice for each experimental group. The level of significance was set at *p < 0.05

**Table 2:** Morphology and contractile properties of *extensor digitorum longus* muscles

Measure	Placebo	Lovastatin
Muscle mass; (mg)	11,89 ± 0,76	12,71 ± 0,10
Cross-sectional area; (μm^2^)	1154,36 ± 115,02	1285,96 ± 64,05
Twitch tension Pt; (g)	4,82 ± 0,37	6,25 ± 0,63 ^*p*=0.088^
Maximal tetanic force P_0_; (g)	28,83 ± 2,23	30,37 ± 2,67
Specific force sP_0_; (g/mg)	2,45 ± 0,16	2,39 ± 0,20
Pt/P_0_	0,17 ± 0,01	0,20 ± 0,01
Time to peak tension TPT; (ms)	27,71 ± 1,54	29,83 ± 2,62
1/2 RT at Pt; (ms)	14,43 ± 1,14	17,00 ± 1,00

The treatment with lovastatin [50 mg/kg/day] for 28 days had the tendency (0,05 < *p* < 0,09) to increase the twitch tension (Pt) of EDL muscles. Data are expressed as means ± SE, *n* = 8 mice for each experimental group. The level of significance was set at *p < 0.05

**Fig. 2:**
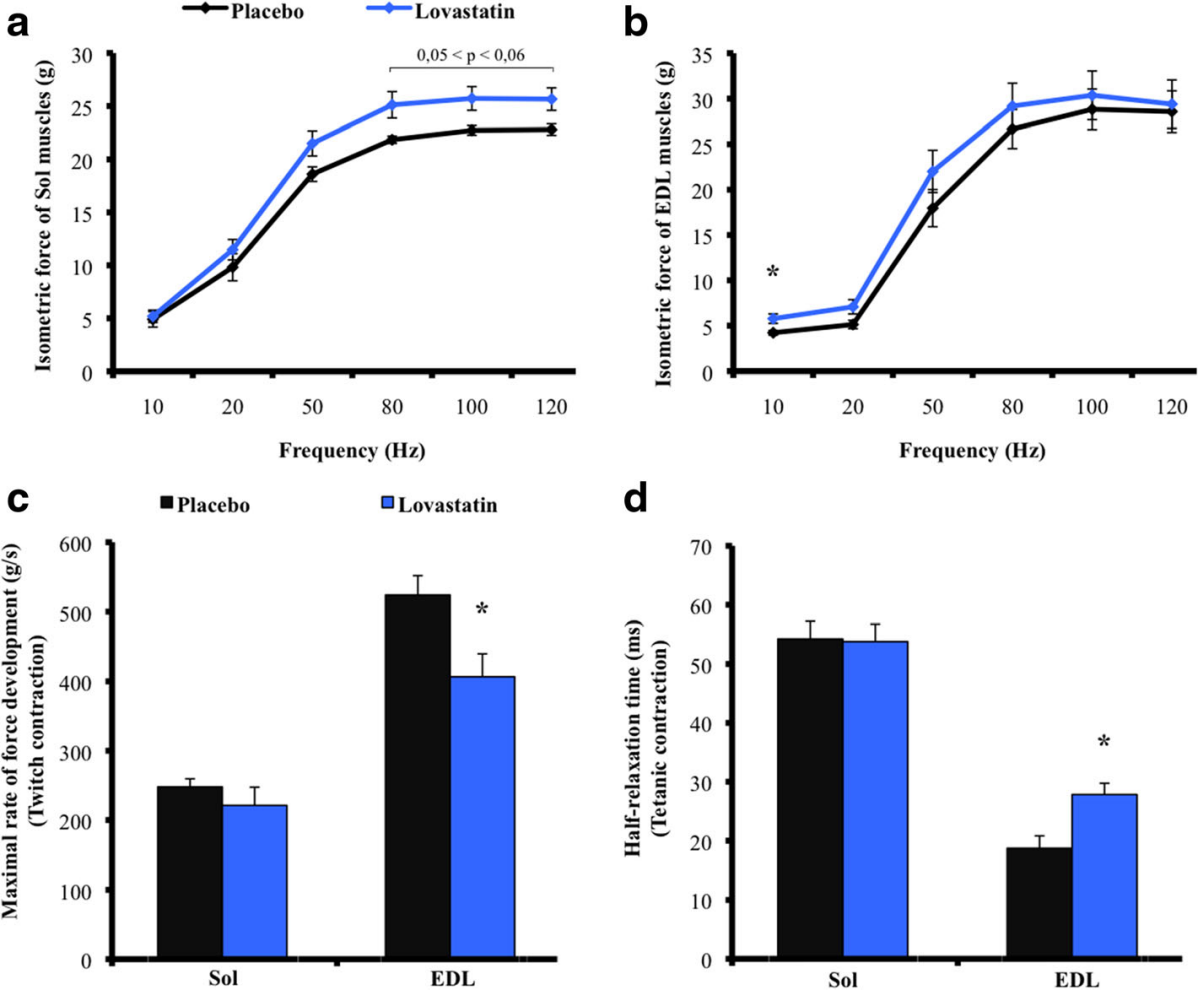
Contractile properties of *soleus* and *extensor digitorum longus* muscles in placebo and lovastatin-treated mice. Frequency-force relationships for Sol (**a**) and EDL muscles (**b**). Maximal rate of force development of twitch contractions (**c**) and half-relaxation time of tetanic contractions (**d**) in Sol or EDL muscles following placebo or lovastatin [50 mg/kg/day] treatments for 28 days. The absolute force production of the Sol muscles from the statin-treated mice tended to increase (*p* < 0.06) when stimulated at 50, 80, 100 and 120 Hz. The treatment significantly decreased the maximal rate of tension generation (dP/dT) for twitch contractions (Pt) by 22.4 % and significantly prolonged the ½ RT for tetanic contractions (P_0_) by 48.7 % in fast-twitch EDL muscles from statin-treated mice compared to EDL muscles from the control mice. Data are expressed as means ± SE, *n* = 8 for each experimental group. The level of significance was set at *p < 0.05

To characterize the impact of lovastatin on muscle function, we measured the ex vivo contractile properties of the Sol and EDL muscles. The control and statin-treated slow-twitch Sol muscles had similar contraction magnitude and kinetics values (Table 1, Fig. 2a, c, d). The absolute force production of the Sol muscles from the statin-treated mice tended to increase (*p* < 0.06) when stimulated at 50, 80, 100 and 120 Hz (Fig. 2a). However, when the force production was normalized with mass, the Sol muscles from the control and statin-treated mice displayed similar levels of specific tetanic force production (Table 1). The lovastatin treatment did not affect the TPT, Pt/P_0_, or Pt ½RT values (Table 2) of fast-twitch EDL muscles. However, the treatment significantly decreased the maximal rate of tension generation (dP/dT) for twitch contractions (Pt) by 22.4 % (Fig. 2c) and significantly prolonged the ½ RT for tetanic contractions (P_0_) by 48.7 % in fast-twitch EDL muscles from statin-treated mice compared to EDL muscles from the control mice (Fig. 2d). In addition, the force production at 10 Hz of EDL muscles from the statin-treated mice was significantly higher (36.7 % gain) than EDL muscles from the control mice (Fig. 2b). Despite the tendency for force to increase at 1 and 20 Hz (*p* < 0.06 statistical trend), there were no significant differences at higher stimulation frequencies (50–120 Hz) (Fig. 2b).

Since these changes in the contractile profiles were suggestive of changes in muscle phenotype, we quantified the percentage of fast MyHC isoform. The changes in the contractile profile of EDL muscles were not associated with significant changes in the proportion of fibers expressing fast MyHC isoform (98 % fast MyHC in EDL muscles) following the lovastatin treatment (data not shown).

## Discussion

SIM has major social and economic consequences. The lack of understanding of how statins impair muscle function at the molecular, cellular, and physiological levels creates numerous barriers to effective treatment [2]. SIM can result in the discontinuation of treatment, leaving specific patients at risk of potential adverse cardiovascular events [43]. The onset of SIM is most likely multifactorial, resulting in multiple physiological impairments in patients with ill-defined musculoskeletal conditions. We showed that a short-term lovastatin treatment induces subtle but significant changes in the contractile profile of fast-twitch EDL muscles.

We also showed that a short-term 28-day lovastatin treatment did not affect the morphology, integrity, or maximal force output of slow-twitch Sol and fast-twitch EDL muscles. However, the force production of subtetanic contractions at 10 Hz was significantly higher while the ½ RT at P_0_ of EDL muscles was prolonged in statin-treated mice compared to control mice. From a physiological standpoint, a prolonged ½ RT at P_0_ should favor a longer lasting Ca^2+^ transient, leading to a gain of force production at a low frequency stimulation (10 Hz). Furthermore, the prolonged ½ RT in fast-twitch EDL muscles also suggested that Ca^2+^ reuptake by the SR is impaired. Ca^2+^ reuptake is almost exclusively mediated by SERCA-1a in fast-twitch fibers. SERCA is critical for Ca^2+^ homeostasis and reuptake by the SR following contractions, allowing cytosolic Ca^2+^ concentrations to return to baseline levels and enabling muscle relaxation [19, 44]. Consistent with our results and a role for statins in the regulation of Ca^2+^ handling, chronic 2-month-long treatments of rats with fluvastatin [20 mg/kg] increase the resting cytosolic Ca^2+^ concentration of EDL muscle fibers by 60 % but have no impact on muscle integrity and grip strength [21]. Pierno et al. (1999) also reported that EDL muscles from simvastatin-treated rats require less depolarization to contract, suggesting that they contain higher levels of cytosolic Ca^2+^ [45]. Liantonio et al. (2007) proposed that mitochondria are responsible for the earlier event, with a subsequent and larger Ca^2+^ leakage from SR stores, and that extracellular Ca^2+^ channels play a negligible role in the increase in cytosolic Ca^2+^. Applying simvastatin to human skeletal muscle fibers triggers a wave of cytosolic Ca^2+^ originating from the SR Ca^2+^ store, while ryanodine and SERCA inhibitors almost completely abolish the increase in cytosolic Ca^2+^ [46]. These findings indicated that the loss of Ca^2+^ homeostasis associated with SIM may be caused by the release of Ca^2+^ from mitochondria and/or the SR store [21, 22, 46]. The prolonged ½ RT and gain of force at a low stimulation frequency provide additional support for the notion that Ca^2+^ handling is impaired, especially in fast-twitch muscles, following statin treatments.

We also showed that the maximum dP/dT, i.e. the peak rate of force development, is 22.4 % lower in EDL muscles from statin-treated mice. A decrease in dP/dT should be associated with a change in muscle phenotype from fast-to slow-twitch. In rats, a simvastatin-treatment induces a 15 % shift from the fast MyHC IIb/x to the slower MyHC IIa phenotype, which is associated with a loss of power output (−41 %) and a reduced shortening velocity (−23 %), with no change in isometric force or CK levels [47]. However, we did not detect any phenotypic changes in EDL muscles after the 28-day lovastatin treatment. Since cholesterol plays a pivotal role in the composition, fluidity, and integrity of the cell membrane, it is possible that statin treatments alter the compliance/rigidity of the muscle membrane, thus decreasing dP/dT. In other words, the contractile component shortens and stretches the elastic component (membrane, extracellular matrix, tendon) by the same amount during contractions. A more compliant membrane/extracellular matrix in statin-treated mice would lower dP/dT. Accordingly, statins cause a deterioration of the biomechanical properties of the Achilles tendon [48] and may be a risk factor for muscle and tendon ruptures and tendinopathies [49, 50]. Statins have thus pleiotropic properties, but the exact mechanism by which they decrease dP/dT requires further investigation.

## Conclusions

Our results provide additional support for the observation that statin treatments have an impact on fast-twitch muscles but have no effect on the function of slow-twitch muscles. Fast-twitch myofibers are also more vulnerable to dysfunction in several forms of muscle disease [51–53]. The changes in the contractile profiles of EDL muscles have also been observed in young and healthy mice treated with a physiological dose of statin. Statins are often prescribed to elderly patients, who are often sarcopenic and undergo a gradual change toward a slow-myofiber phenotype [54, 55]. Would these changes in contractile properties be greater in patients with chronic conditions? Would this shift toward slow phenotype increase the risk of falls given that explosive bursts of strength and power are needed to prevent falls? In this context, it would be relevant to determine whether statin treatments exacerbate aging-induced muscle dysfunction and whether the prevalence of SIM is higher in patients with chronic physical conditions. Further animal and human investigations are warranted to determine how statins induce SIM and muscle dysfunction.

## Abbreviations

½ RT: Half relaxation time
CK: Creatine kinase
CSA: Cross-sectional aera
dP/dT: Maximal rate of force development
EDL: Extensor digitorum longus
H&E: Hematoxylin & eosin
L_0_: Optimal lenght
MyHC: Myosin heavy chain
P_0_: Maximum tetanic tension
Pt: Twitch tension
SERCA: Sarco-endoplasmic reticulum calcium ATPase
SIM: Statin-induced myopathy
Sol: soleus
SR: Sarcoplasmic reticulum
TPT: Time to peak tension
